# Supplementary material for: Joint Registration and Conformal Prediction for Partially Observed Functional Data
Source: J Comput Graph Stat. Author manuscript; Available in PMC 2026 May 12. (PMC13160368; doi:10.1080/10618600.2026.2634823)
Supplement: Supp 1 [file NIHMS2168257-supplement-Supp_1.zip › ucgs_a_2634823_sm2285.pdf]

Joint Registration and Conformal Prediction for Partially Observed Functional Data

Fangyi Wang, Sebastian Kurtek and Yuan Zhang
Department of Statistics, The Ohio State University

A Lemmas and proofs

Exchangeability lemmas. We state and prove three exchangeability lemmas that are used to establish the coverage guarantee of PIs generated using `FFCP`, `SFCP` and `SFCPP`.

Lemma 1 (Exchangeability, `FFCP`). *Suppose Assumption 1 holds. For any $t \in \mathcal{T}$, let $X_i = f_i^{\mathcal{J}}$ and $Y_i(t) = f_i(t)$ for $i = 1, \ldots, n+1$. Then $\{(X_i, Y_i(t))\},\ i = 1, \ldots, n+1$ are exchangeable.*

Lemma 2 (Exchangeability, `SFCP`). *Suppose Assumption 1 holds. For any $t \in \mathcal{T}$, let $X_i = f_{n_1+i}^{\mathcal{J}}$ and $Y_i(t) = \tilde{f}_i(t) = (f_{n_1+i} \circ \gamma_i^*)(t)$ for $i = 1, \ldots, n_2+1$. Then $\{(X_i, Y_i(t))\},\ i = 1, \ldots, n_2+1$ are exchangeable.*

Lemma 3 (Exchangeability, `SFCPP`). *Suppose Assumption 1 holds. Let $X_i = f_{n_1+i}^{\mathcal{J}}$ and $\mathbf{Y}_i = (\gamma_i^*(t_1), \ldots, \gamma_i^*(t_T))$ for $i = 1, \ldots, n_2+1$. Then $\{(X_i, \mathbf{Y}_i)\},\ i = 1, \ldots, n_2+1$ are exchangeable.*

Proof. We first prove Lemma 2. Let $\bar{f}$ be the sample Karcher mean of functions $f_1, \ldots, f_{n_1}$ in the training set. For any $t \in \mathcal{T}$, define mapping $g : \mathcal{F} \times \mathcal{F} \times \mathbb{R} \to \mathcal{X} \times \mathcal{Y},\ g(f_{n_1+i}, \bar{f}, U) = (X_i, Y_i(t)),\ i = 1, \ldots, n_2+1$. Given U, $\bar{f}$ and f_{n_1+i}, g is a deterministic procedure, which involves (i) truncating f_{n_1+i} at $t = U$ to generate X_i, (ii) registering f_{n_1+i} to $\bar{f}$, and (iii) evaluating the registered f_{n_1+i} at $t \in \mathcal{T}$; steps (ii) and (iii) result in $Y_i(t),\ t \in \mathcal{T}$. Under Assumption 1, g is symmetric with respect to $f_{n_1+i},\ i = 1, \ldots, n_2+1$. Further, again using Assumption 1, for any $t \in \mathcal{T}$, $(X_i, Y_i(t))|(\bar{f}, U),\ i = 1, \ldots, n_2+1$ are i.i.d., which implies $(X_i, Y_i(t)),\ i = 1, \ldots, n_2+1$ are exchangeable.

Proofs of Lemmas 1 and 3 follow similar arguments as the proof of Lemma 2. For Lemma 1, FFCP only involves truncating $\{f_i\}$ at $t = U$ to generate X_i and evaluating $\{f_i\}$ at $t \in \mathcal{T}$ to generate $Y_i(t)$ for $i = 1, \ldots, n+1$. Thus, under Assumption 1, $(X_i, Y_i(t))|U,\ i = 1, \ldots, n+1$ are i.i.d.. For Lemma 3, and again using Assumption 1, since registering $f_{n_1+i},\ i = 1, \ldots, n_2+1$ to $\bar{f}$ is deterministic and symmetric, the resulting warping functions $\gamma_i^*|\bar{f},\ i = 1, \ldots, n_2+1$ are i.i.d., and thus $\mathbf{Y}_i|\bar{f}$ and $(X_i, \mathbf{Y}_i)|(\bar{f}, U),\ i = 1, \ldots, n_2+1$ are also i.i.d. □

Proof of Theorem 1. Lemma 2 ensures exchangeability of $\{(X_i, Y_i(t))\},\ i = 1, \ldots, n_2+1$. Given exchangeable predictors and responses, a permutation symmetric algorithm, and absolute residual as nonconformity score, the coverage guarantee in Theorem 1 follows directly from standard conformal prediction literature [Vovk et al., 2005, Foygel Barber et al., 2023]. The anti-conservative bound follows from the proof of Theorem 2 in Lei et al. [2018], assuming no ties in $\{S_i(t)\}$. □

B Additional simulations

B.1 Choice of distance metric and bandwidth parameter for SFCP

We compare three distances for neighborhood smoothing, $d_2(\cdot,\cdot)$, $d_{\mathrm{FR}}(\cdot,\cdot)$ and $d_a(\cdot,\cdot)$, and two bandwidth tuning methods, global and local. We assess performance using data from a homogeneous population and a heterogeneous population. Data from the homogeneous population is simulated as in the main article. For the heterogeneous population, we simulate one- and two-peak functions: one-peak functions are given by $f_i(t) = Z_i \exp\{-(t-0.5)^2/0.25\},\ Z_i \overset{iid}{\sim} N(2, 0.1)$, while two-peak functions are the same as in the homogeneous population. We use $\mathcal{H} = \{\mathcal{Q}_\beta(\{D_{i,j}\}_{1\le i<j\le n+1}),\ \beta = 0.1, \ldots, 0.9\}$. Figure 1 shows the results. Rows 1 & 2 (3 & 4) use functions from a homogeneous (heterogeneous) population with phase variation. Rows 1 & 3 (2 & 4) use global (local) tuning for h. Panel (a) shows

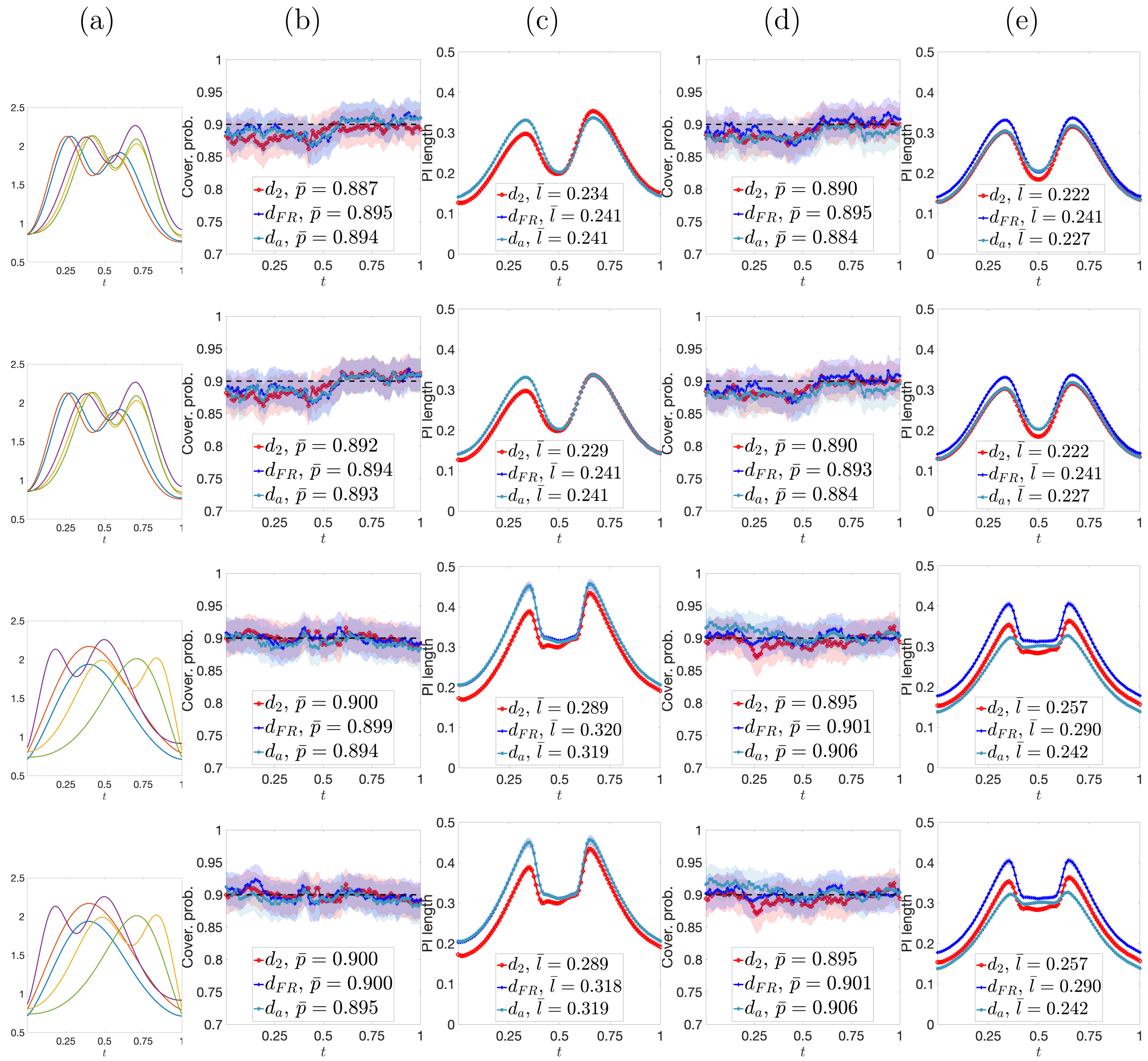

Figure 1: Row 1: homogeneous population, global tuning. Row 2: same as 1, but with local tuning. Row 3: heterogeneous population, global tuning. Row 4: same as 3, but with local tuning. (a) Random subsample of 5 functions. (b) Coverage rates (solid) with 95% CIs (shaded regions), $U = 0.25$. (c) Average PI lengths, $U = 0.25$. (d) Same as (b), but $U = 0.75$. (e) Same as (c), but $U = 0.75$. In (b)-(e), $d_2(\cdot,\cdot)$ is in red, $d_{FR}(\cdot,\cdot)$ in dark blue and $d_a(\cdot,\cdot)$ in light blue.

a random subsample of five functions for each case. Panels (b)-(e) compare pointwise coverage rates (with 95% CIs as shaded regions) and average PI lengths for truncation time points $U = 0.25$ ((b) & (c)) and $U = 0.75$ ((d) & (e)) in f_{n+1}, respectively. Our method provides valid coverage in all cases. When $U = 0.25$, using $d_2(\cdot,\cdot)$ yields the

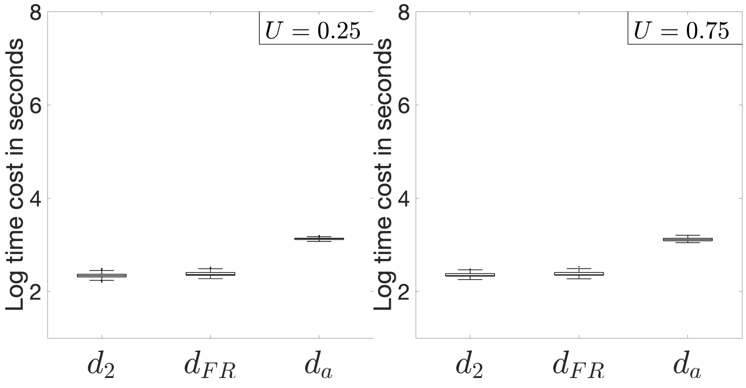

Figure 2: Log time cost in seconds for a single prediction for (a) $U = 0.25$ and (b) $U = 0.75$.

smallest time-averaged PI length $\bar{\ell}$. On the other hand, when functions are sampled from a heterogeneous population and $U = 0.75$ (rows 3 & 4(e)), $d_a(\cdot,\cdot)$ yields PIs with smallest $\bar{\ell}$. This is because, when a larger portion of the predictors is observed, $d_a(\cdot,\cdot)$ is effective at registering their geometric features, resulting in better predictive power.

To assess computational efficiency, Figure 2 shows log time cost in seconds for a single prediction using the three distances. As expected, $d_2(\cdot,\cdot)$ and $d_{FR}(\cdot,\cdot)$ are faster than $d_a(\cdot,\cdot)$. Summarily, using $d_2(\cdot,\cdot)$ is most computationally efficient and results in best prediction accuracy in most cases. Further, local and global bandwidth tuning result in valid coverage rates, comparable PI lengths and computational cost. Thus, we use $d_2(\cdot,\cdot)$ with local tuning in all subsequent simulations and all simulations in the main paper except for prediction of relative phase, which uses global tuning. See Algorithm 4 in Section C.2 for a detailed bandwidth tuning algorithm.

B.2 Comparison of computational cost

To compare computational efficiency of `SoF`, `FoF`, `SFCP` and `FFCP`, we recorded log time cost in seconds for a single prediction of a target function based on $n = 100$ complete functions sampled at $T = 100$ time points. For `SFCP`, we used an equal number of training and calibration samples, i.e., $n_1 = n_2 = 50$. Figure 3 shows the results for data without (a) and with (b) phase variation. In general, `FoF` is faster than other methods. This is

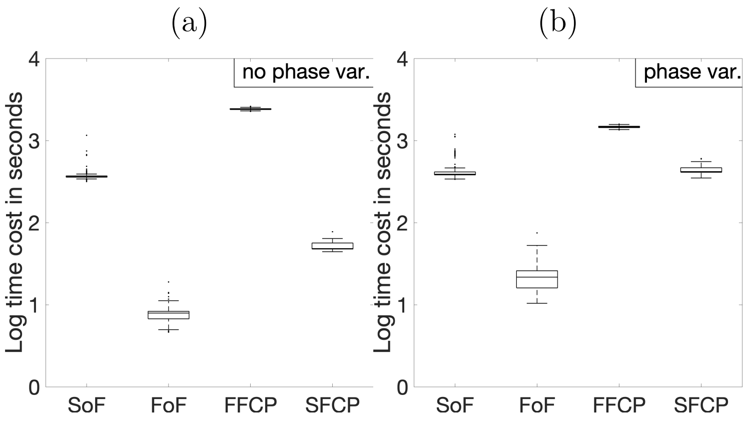

Figure 3: (a) & (b) Log time cost in seconds for a single prediction for functions without and with phase variation, respectively.

because `FoF` performs prediction for f_{n+1} simultaneously at all time points. In contrast, the other methods predict the target function in a pointwise manner. Note that computational efficiency of the pointwise approaches can be improved via a parallel implementation across time points. Also, `SFCP` is faster than `FFCP` in both cases. Unlike standard split conformal methods, `SFCP` does not avoid the grid search. However, the computational cost depends on the number of predictor-response pairs. `SFCP` only uses $n_2 = 50$ samples in the calibration set to generate $\{X_i, Y_i(t)\}$ for prediction, which is much smaller than the $n = 100$ samples used in `FFCP`. `SFCP` is slower when phase variation is present in the data than when it is not. The Karcher mean is estimated via an iterative algorithm, which requires more iterations to converge in the presence of phase variation.

B.3 Sensitivity analyses

Simulation S1: Effects of data pre-smoothing. In some real-world data scenarios, it is desirable to mitigate the effects of noise on prediction by applying smoothing. Thus, to evaluate the effect of functional data smoothing on coverage validity and prediction accuracy of `SFCP`, we consider two methods: (i) projection onto ten Fourier basis functions, and (ii) moving average smoothing with window size of 12. For this simulation, we added

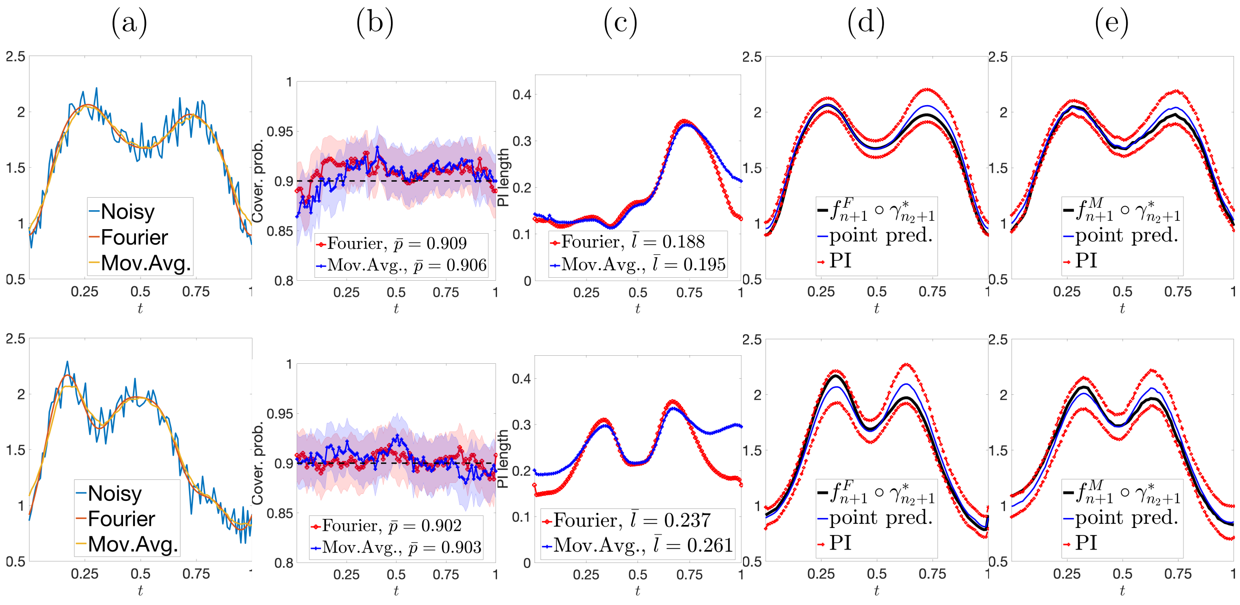

Figure 4: `SFCP` for smoothed functions, $U = 0.5$. Row 1: without phase variation. Row 2: with phase variation. (a) Noisy observation (blue), smoothed function after applying Fourier basis projection (red) and moving average smoother (yellow). (b) & (c) Coverage rates (solid) with 95% CIs (shaded regions) and average PI lengths (Fourier basis projection in red and moving average smoother in blue), respectively. (d) & (e) Target function (black), point prediction (blue) and pointwise PIs (red) after applying Fourier basis projection and moving average smoother, respectively.

i.i.d. pointwise Gaussian noise to each function from the homogeneous population (with and without phase variation), i.e., $\epsilon_i(t) \overset{i.i.d.}{\sim} N(0, 0.01),\ i = 1, \dots, n+1,\ t = 1, \dots, T$. After adding noise, we generate the partial function using a truncation time point $U = 0.5$. To maintain exchangeability, we apply smoothing in the following manner. First, we construct the predictors $\{X_i\}$ as before. Then, we separately smooth the predictors and the complete functions resulting in $\{X_i^F\}$ and $\{f_i^F\}$ for Fourier basis projection, or $\{X_i^M\}$ and $\{f_i^M\}$ for the moving average smoother; these then serve as inputs into `SFCP`. Thus, the prediction target becomes $f^F_{n+1} \circ \gamma^*_{n_2+1}$ or $f^M_{n+1} \circ \gamma^*_{n_2+1}$, respectively, where $\gamma^*_{n_2+1}$ registers f^F_{n+1} or f^M_{n+1} to the sample Karcher mean calculated using pre-smoothed training data $f^F_1, \dots, f^F_{n_1}$ or $f^M_1, \dots, f^M_{n_1}$. Exchangeability is preserved for $\left(X_i^F, Y_i^F(t)\right)$ (or $\left(X_i^M, Y_i^M(t)\right)$), where

$Y_i^F(t) = (f_{n_1+i}^F \circ \gamma_i^*)(t)$ (or $Y_i^M(t) = (f_{n_1+i}^M \circ \gamma_i^*)(t)$), since the smoothing procedure is performed independently and symmetrically for each X_i and f_i.

Figure 4(a) shows a randomly sampled noisy functional observation (blue) and its smoothed versions (Fourier basis projection in red and moving average smoother in yellow). Panels (b) & (c) show pointwise coverage rates (with 95% CIs as shaded regions) and average PI lengths, respectively. Panels (d) & (e) show examples of PIs for $f_{n+1}^F \circ \gamma_{n_2+1}^*$ and $f_{n+1}^M \circ \gamma_{n_2+1}^*$, respectively, with a point prediction in blue (midpoint of PIs at each time point). `SFCP` yields PIs with valid coverage in all cases. The PIs constructed after Fourier basis projection are shorter close to $t = 0$ and $t = 1$ than those generated after applying the moving average smoother. This is not surprising due to the boundary effect associated with the latter approach. Overall, the time averaged PI length $\bar{\ell}$ is a bit smaller for Fourier basis projection. Finally, both methods yield prediction bands that are effective at capturing the geometric features, i.e., two peaks and one valley, of the underlying noiseless function.

Simulation S2: Effects of different truncation time points. We fix the truncation time point $U = 0.5$ in most simulated and real-world data examples in the main article. Here, we evaluate the performance of `SFCP` for different choices of truncation time points, by either fixing $U = 0.1, 0.3, 0.7, 0.9$ or sampling $U \sim \text{Unif}(0.1, 0.9)$ in each Monte Carlo sample. The results are shown in Figure 5, where panels (a)-(e) correspond to $U = 0.1, 0.3, 0.7, 0.9$ and random, respectively. Rows 1 & 2 show pointwise coverage rates (95% CIs as shaded regions) and average PI lengths, respectively, using data from a homogeneous population without (blue) and with (yellow) phase variation. `SFCP` maintains coverage validity in all cases. When there is no phase variation in the data, observing a larger proportion of f_{n+1} results in smaller average PI lengths (from $\bar{\ell} = 0.149$ when $U = 0.1$ to $\bar{\ell} = 0.086$ when $U = 0.9$). When phase variation is present in the data, the PI lengths are comparable across different truncation time points. This indicates that the amount of f_{n+1} that is observed has little effect on the prediction accuracy for the ampli-

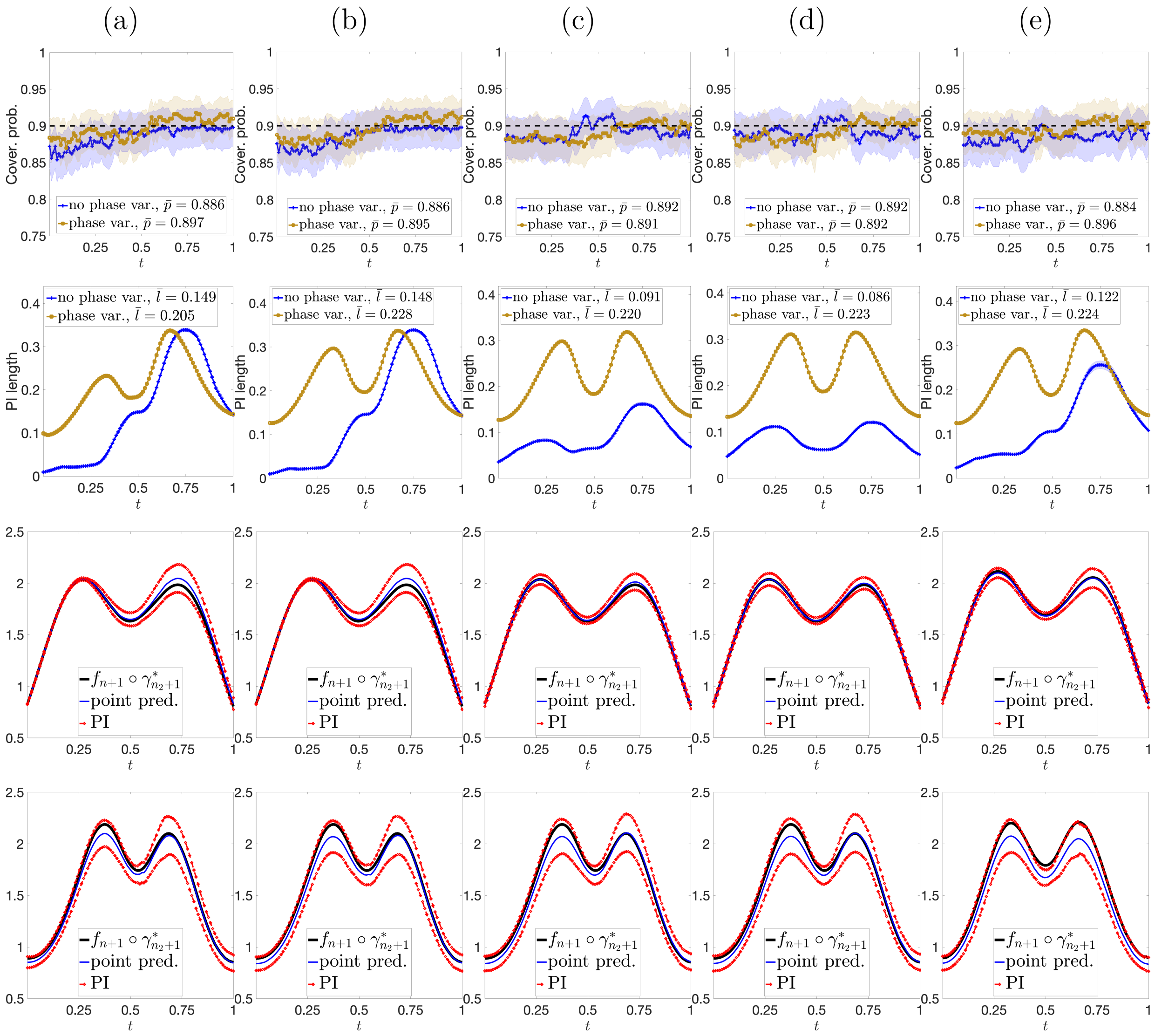

Figure 5: (a)-(d) Truncation time point $U = 0.1, 0.3, 0.7, 0.9$, respectively. (e) Truncation time point $U \sim \text{Unif}(0.1, 0.9)$. Rows 1 & 2: coverage rates (solid) with 95% CIs (shaded regions) and average PI lengths for data without (blue) and with (yellow) phase variation. Rows 3 & 4: target function (black), point prediction (blue), pointwise PIs (red) for data without and with phase variation, respectively.

tude of f_{n+1} in the presence of phase variation. Rows 3 & 4 show examples of PIs (red) and point predictions (blue) for a target function (black) using data without and with phase variation, respectively. In the absence of phase variation, the uncertainty for predicting the size of the second peak decreases as we observe a larger proportion of f_{n+1}, which matches

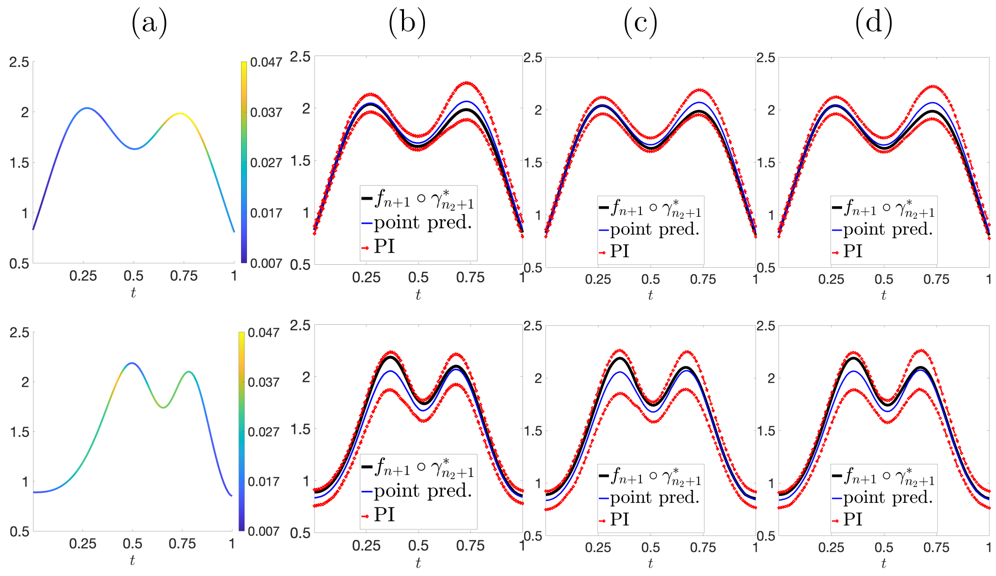

Figure 6: Row 1: without phase variation. Row 2: with phase variation. (a) f_{n+1} colored by sample standard deviation of pointwise PI lengths. (b)-(d) Target function (black), point prediction (blue) and pointwise PIs (red) for three different data splits into training and calibration sets.

the trend in the PI lengths. The prediction bands are much more similar across truncation time points when there is phase variation. To summarize, `SFCP` provides valid and accurate pointwise PIs when a reasonable portion (e.g., 10%) of the partial function is observed.

Simulation S3: Effects of different data splits into training and calibration sets. As part of `SFCP`, we first randomly split the data into training and calibration sets. The resulting pointwise PIs for the amplitude of f_{n+1} depend on the sample Karcher mean $\bar{f}$ of the training set, which can be different across the $\binom{n}{n_1}$ possible data splits. To examine the effects of this procedure on the performance of `SFCP`, we consider 500 random splits of $f_1, \ldots, f_n$ while keeping f_{n+1} unchanged. Panel (a) in Figure 6 shows f_{n+1} colored by the sample standard deviation of pointwise PI lengths for data without (row 1) and with (row 2) phase variation. Panels (b)-(d) show examples of the target function (black), point prediction (blue) and pointwise PIs (red) for three different random splits. Notice that the prediction target $f_{n+1} \circ \gamma^*_{n_2+1}$, i.e., the amplitude of f_{n+1}, is different in each case, since

the relative phase of f_{n+1} with respect to $\bar{f}$ changes for different splits. In (a), the sample standard deviations of PI lengths range from 0.007 to 0.047 across the time domain and are relatively small compared to the overall magnitude of the PI lengths. When there is no phase variation, PI lengths vary the most (standard deviation > 0.04) around the second peak of the function. This is supported by row 1 in Figure 6(b)-(d), where the prediction bands have very similar widths except for the sizes of the second peak. On the other hand, when phase variation is present in the data, the PI lengths have smaller standard deviations, with a maximum of 0.0414 before the first peak. In row 2, Figure 6(b)-(d), the differences in the width and shape of the prediction bands are visually negligible. These results suggest that `SFCP` is robust to the procedure of random data splitting into training and calibration sets.

B.4 Comparisons to Diquigiovanni et al. [2022b]

In addition to `SoF` and `FoF`, we provide a comparison to the method proposed by Diquigiovanni et al. [2022b], which applies conformal prediction to multivariate functional responses with domain adaptive nonconformity scores using a modulation function. The resulting conformal prediction band has valid overall coverage for the entire function. Their framework can be applied using any functional regression framework for a multivariate functional response that satisfies permutation symmetry. To compare our results to this approach, we use the `R` package `conformalInference.fd` [Diquigiovanni et al., 2022a]. The partial functions $f_i^{\mathcal{J}}$ serve as predictors and the complete functions f_i are the responses. We consider the example in Section 4, Simulation 1, and the real-world data examples in Section 5, Example 1. We refer to their method as `CPB-FD` (Conformal Prediction Bands for multivariate Functional Data). The results are shown in Figure 7. The first row shows pointwise coverage rates (with 95% CIs as shaded regions) and examples of PIs (red) and a point prediction (blue) for simulated two-peak functions without (panels (a)&(b)) and with

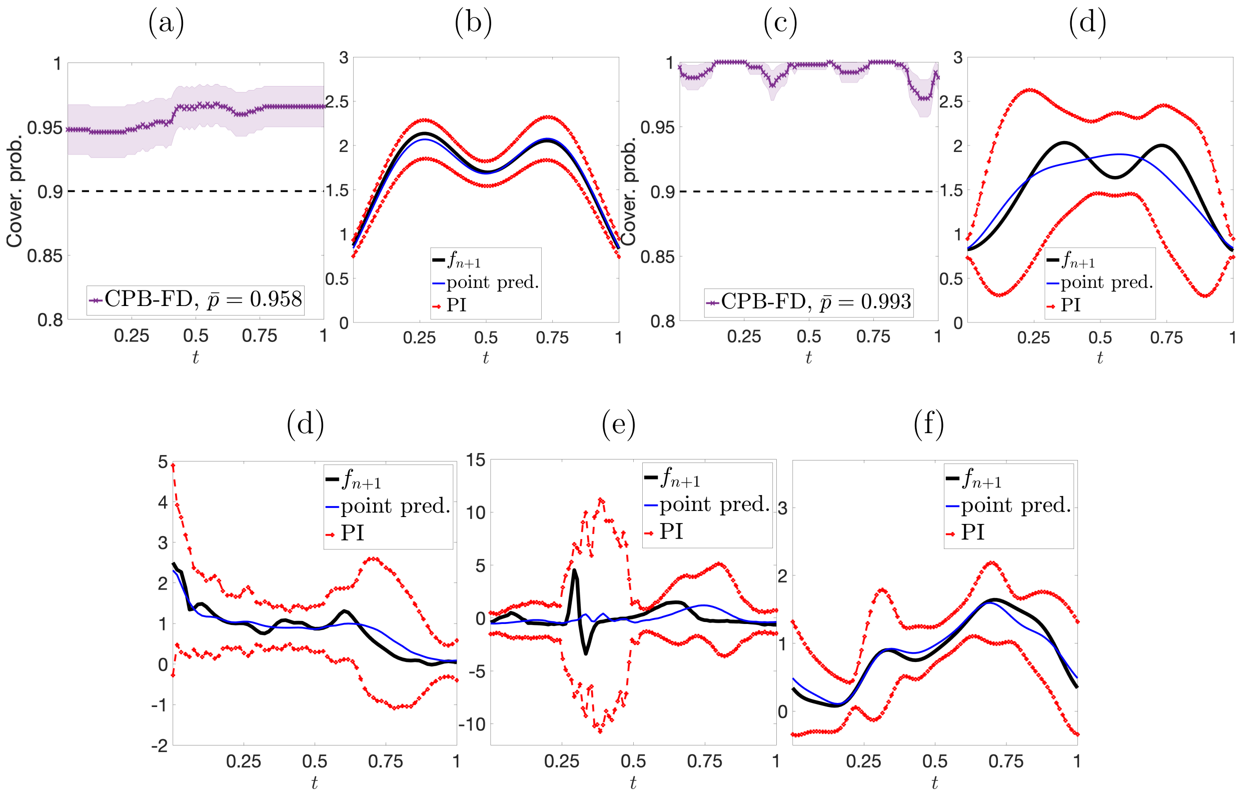

Figure 7: Prediction results using CPB-FD. (a) Pointwise coverage rates (solid) with 95% CIs (shaded regions) for simulated two-peak functions without phase variation, computed using $B = 500$ Monte Carlo iterations. (b) Ground truth f_{n+1} (black), point prediction (blue) and pointwise PIs (red) for simulated two-peak functions without phase variation. (c)&(d) Same as (a)&(b), but for simulated two-peak functions with phase variation. (d)-(f) Ground truth f_{n+1} (black), point prediction (blue) and pointwise PIs (red) for three real-world data examples: Berkeley growth rate functions, PQRST complexes and traffic flow rate functions, respectively.

(panels (c)&(d)) phase variation. The overall coverage rates (with 95% CIs) for data without and with phase variation are 0.896 $(0.883, 0.909)$ and 0.932 $(0.921, 0.943)$, respectively. `CPB-FD` yields prediction bands with valid overall coverage, and thus pointwise coverage validity is automatically satisfied. However, when phase variation is present in the data, the predictions tend to be overly conservative (row 1, panel (d)). Row 2 shows results based on real-world data examples, where we also observe conservative prediction bands due to underlying phase variation. To summarize, `CPB-FD` has valid overall coverage, but

is overly conservative when functional data contains phase variation.

C Algorithms

C.1 Main algorithms with fixed bandwidth parameter

Algorithm 1 Full Functional Conformal Prediction (`FFCP`)

Input: Data $f_1, \ldots, f_n$, $f_{n+1}^{\mathcal{J}}$ for $\mathcal{J} = [0, U]$; significance level α;

length of time grid T; search grid resolution M and range $[a, b]$.

For neighborhood smoothing: kernel function $K(\cdot)$; bandwidth h; distance $d(\cdot, \cdot)$.

Output: $1 - \alpha$ conformal prediction sets $\mathcal{I}_{t_k}$ for each $t_k \in \mathcal{T}$.

1. Set $X_i = f_i^{\mathcal{J}}$ for $i = 1, \ldots, n+1$.

for k in 1:T **do**

2. Set $t_k = (k-1)/(T-1)$, $Y_i(t_k) = f_i(t_k)$ for $i = 1, \ldots, n$.

3. Initialize $\mathcal{I}_{t_k} = \emptyset$.

4. Construct search grid over $[a, b]$ with M points.

for each candidate y in the search grid **do**

5. Set $Y_{n+1}(t_k) = y$, compute $\widehat{Y_1(t_k)}, \ldots, \widehat{Y_{n+1}(t_k)}$ using neighborhood smoothing.

6. Compute nonconformity scores $S_i(t) = |Y_i(t_k) - \widehat{Y_i(t_k)}|$ for $i = 1, \ldots, n+1$.

7. Include y in $\mathcal{I}_{t_k}$ if $S_{n+1}(t) \leq \mathcal{Q}_{1-\alpha}(\{S_1(t), \ldots, S_{n+1}(t)\})$.

end for

end for

Return $\{\mathcal{I}_{t_k}\}_{k=1}^{T}$.

Algorithm 2 Split Functional Conformal Prediction (`SFCP`)

Input: Data $\mathcal{D} = \{f_1, \ldots, f_n\}$, $f_{n+1}^{\mathcal{J}}$ for $\mathcal{J} = [0, U]$; significance level α; number of samples in training set, n_1; length of time grid T; search grid resolution M and range $[a, b]$.

For neighborhood smoothing: kernel function $K(\cdot)$; bandwidth h; distance $d(\cdot, \cdot)$.

Output: $1 - \alpha$ conformal prediction sets $\mathcal{I}_{t_k}$ for each $t_k \in \mathcal{T}$.

1. Randomly split $f_1, \dots, f_n$ into training set $\mathcal{D}_{tr} = \{f_1, \dots, f_{n_1}\}$ and calibration set $\mathcal{D}_{cal} = \{f_{n_1+1}, \dots, f_n\}$, set $n_2 = n - n_1$.

2. Compute the Karcher mean $\bar{f}$ of training set $f_1, \dots, f_{n_1}$.

3. **for** $i = 1$ to n_2 **do** Register $f_{n_1+i} \in \mathcal{D}_{\text{cal}}$ to $\bar{f}$ and get γ_i^*. Set $\tilde{f}_i = f_{n_1+i} \circ \gamma_i^*$. **end for**

4. Set $X_i = f_{n_1+i}^{\mathcal{J}}$ for $i = 1, \dots, n_2 + 1$.

for k in 1:T **do**

5. Set $t_k = (k-1)/(T-1)$, $Y_i(t_k) = \tilde{f}_i(t_k)$ for $i = 1, \dots, n_2$.

6. Initialize $\mathcal{I}_{t_k} = \emptyset$.

7. Construct search grid over $[a, b]$ with M points.

for each candidate y in the search grid **do**

8. Set $Y_{n_2+1}(t_k) = y$ and compute $\widehat{Y_1(t_k)}, \dots, \widehat{Y_{n_2+1}(t_k)}$ using neighborhood smoothing.

9. Compute nonconformity scores $S_i(t) = |Y_i(t_k) - \widehat{Y_i(t_k)}|$ for $i = 1, \dots, n_2 + 1$.

10. Include y in $\mathcal{I}_{t_k}$ if $S_{n_2+1}(t) \leq \mathcal{Q}_{1-\alpha}(\{S_1(t), \dots, S_{n_2+1}(t)\})$.

end for

end for

Return $\{\mathcal{I}_{t_k}\}_{k=1}^{T}$.

Remark. *For the other observational regimes (fragmented or sparse) discussed in Section 3.2 in the main article, Step 4 in Algorithm 2 needs to be modified accordingly based on the definition of the predictors X_i. Also, the distance $d(\cdot, \cdot)$ between predictors for neighborhood smoothing used in Step 8, which is one of the inputs of the algorithm, needs to be chosen to match the fragmented or sparse regime as discussed in Section 3.2 in the main article.*

Algorithm 3 Split Functional Conformal Prediction for Relative Phase (`SFCPP`)

Input: Data $\mathcal{D} = \{f_1, \dots, f_n\}$, $f_{n+1}^{\mathcal{J}}$ for $\mathcal{J} = [0, U]$; significance level α;

number of samples in training set, n_1; length of time grid T; search grid resolution M.

For neighborhood smoothing: kernel function $K(\cdot)$; bandwidth h; distance $d(\cdot, \cdot)$.

Output: $1-\alpha$ conformal prediction set $\mathcal{I}_\gamma$.

1. Randomly split $f_1, \ldots, f_n$ into training set $\mathcal{D}_{tr} = \{f_1, \ldots, f_{n_1}\}$ and calibration set $\mathcal{D}_{cal} = \{f_{n_1+1}, \ldots, f_n\}$, set $n_2 = n - n_1$.

2. Compute the Karcher mean $\bar{f}$ of training set $f_1, \ldots, f_{n_1}$.

3. **for** $i = 1$ to n_2 **do** Register $f_{n_1+i} \in \mathcal{D}_{\text{cal}}$ to $\bar{f}$ and get γ_i^* **end for**

4. Set $X_i = f_{n_1+i}^{\mathcal{J}}$ for $i = 1, \ldots, n_2 + 1$.

5. Set $\mathbf{Y}_i = (\gamma_i^*(t_1), \ldots, \gamma_i^*(t_T)), i = 1, \ldots, n_2$.

6. Initialize $\mathcal{I}_\gamma = \emptyset$.

7. Construct search grid over $[0,1]^{T-2}$ with M points per dimension; retain only vectors $(y_2, \ldots, y_{T-1})$ with $0 < y_2 < \cdots < y_{T-1} < 1$.

for each candidate $\boldsymbol{y} = (0, y_2, \ldots, y_{T-1}, 1)$ in the search grid **do**

8. Set $\mathbf{Y}_{n_2+1} = \boldsymbol{y}$ and compute $\widehat{\mathbf{Y}}_1, \ldots, \widehat{\mathbf{Y}}_{n_2+1}$ using neighborhood smoothing.

9. Compute nonconformity scores $S_i = d_w(\mathbf{Y}_i, \widehat{\mathbf{Y}}_i)$ for $i = 1, \ldots, n_2 + 1$.

10. Include $\boldsymbol{y}$ in $\mathcal{I}_\gamma$ if $S_{n_2+1} \leq \mathcal{Q}_{1-\alpha}(\{S_1, \ldots, S_{n_2+1}\})$.

end for

Return $\mathcal{I}_\gamma$.

C.2 Tuning for bandwidth parameter

Algorithm 4 Tuning for bandwidth parameter in neighborhood smoothing

Input: Tuning method: global or local; main algorithm: one of `FFCP`, `SFCP` or `SFCPP` and their corresponding inputs; candidate bandwidth: fixed values $\mathcal{H} = \{h_1, h_2, \dots\}$ or lower $\beta \in (0,1)$ quantiles $\mathcal{B} = \{\beta_1, \beta_2, \dots\}$.

Output: PIs from main algorithms with optimal bandwidth.

if $\mathcal{H}$ is provided **then skip else** initialize $\mathcal{H} = \emptyset$

for each candidate quantile $\beta \in \mathcal{B}$ **do**

calculate $h = \mathcal{Q}_\beta(\{D_{i,j}\}_{i<j})$, where $D \in \mathbb{R}^{(n_2+1)\times(n_2+1)}$ for `SFCP` and `SFCPP` ($D \in \mathbb{R}^{(n+1)\times(n+1)}$ for `FFCP`) with $D_{i,j} = d(X_i, X_j)$, include h in $\mathcal{H}$.

end for

for each candidate bandwidth $h \in \mathcal{H}$ **do**

Run main algorithm with bandwidth input h, get $\{\mathcal{I}_{t_k}^h\}_{k=1}^T$ for `FFCP` or `SFCP` or $\mathcal{I}_\gamma^h$ for `SFCPP`.

end for

if main algorithm is `SFCPP` **then**

Compute $h^* = \arg\min_{h\in\mathcal{H}} \frac{1}{T-2}\sum_{k=2}^{T-1} \text{length}(\mathcal{I}_{\gamma,k}^h)$, where $\text{length}(\mathcal{I}_{\gamma,k}^h) = \max_{\boldsymbol{y}\in\mathcal{I}_\gamma^h} y_k - \min_{\boldsymbol{y}\in\mathcal{I}_\gamma^h} y_k,\ k = 2, \dots, T-1$.

Return $\mathcal{I}_\gamma^{h^*}$.

else

if tuning method is global, compute $h^* = \arg\min_{h\in\mathcal{H}} \frac{1}{T}\sum_{k=1}^{T} \text{length}(\mathcal{I}_{t_k}^h)$.

Return $\{\mathcal{I}_{t_k}^{h^*}\}_{k=1}^T$.

else compute $h_{t_k}^* = \arg\min_{h\in\mathcal{H}} \text{length}(\mathcal{I}_{t_k}^h),\ k = 1, \dots, T$.

Return $\{\mathcal{I}_{t_k}^{h_{t_k}^*}\}_{k=1}^T$.

end if

References

J. Diquigiovanni, M. Fontana, A. Solari, S. Vantini, P. Vergottini, and R. Tibshirani. *conformalInference.fd: Tools for Conformal Inference for Regression in Multivariate Functional Setting*, 2022a. URL https://CRAN.R-project.org/package=conformalInference.fd. R package version 1.1.1.

J. Diquigiovanni, M. Fontana, and S. Vantini. Conformal prediction bands for multivariate functional data. *Journal of Multivariate Analysis*, 189:104879, 2022b.

R. Foygel Barber, E.J. Candès, A. Ramdas, and R.J. Tibshirani. Conformal prediction beyond exchangeability. *The Annals of Statistics*, 51(2):816–845, 2023.

J. Lei, M. G'Sell, A. Rinaldo, R.J. Tibshirani, and L. Wasserman. Distribution-free predictive inference for regression. *Journal of the American Statistical Association*, 113(523): 1094–1111, 2018.

V. Vovk, A. Gammerman, and G. Shafer. *Algorithmic Learning in a Random World*. Springer, 2005.
