# Supplementary material for: Joint Registration and Conformal Prediction for Partially Observed Functional Data
Source: J Comput Graph Stat. Author manuscript; Available in PMC 2026 May 12. (PMC13160368; doi:10.1080/10618600.2026.2634823)
Supplement: Supp 1 [file NIHMS2168257-supplement-Supp_1.zip › JointRegistrationConformalPrediction_MATLAB2022b.zip!/JointRegistrationConformalPrediction_MATLAB2022b/jdtuck-fdasrvf_MATLAB-3.6.3.0/bspline_tools/doc/html/BsplineDoc.html]

B-spline toolbox 

# B-spline toolbox

Basic toolbox for polynomial B-splines on a regular grid

## Contents

- Introduction
- Installation
- Running the unit tests
- Introductory example
- Approximaton accuracy
- Coefficient characteristics
- Derivatives and integration
- 2D splines

## Introduction

B-splines is a natural signal representation for continous signals, where many continous-domain operations can be carried out exactly once the B-spline approximation has been done.

The B-spline estimation procedure in this toolbox using allpole filters is based on the classic papers by M. Unser and others [1,2,3], it allows very fast estimation of B-spline coefficients when the sampling grid is uniform. Evaluation/interpolation is also a linear filter operation.

The toolbox has two layers; a set of functions for the fundamental operations on polynomial B-splines, and an object-oriented wrapper which keeps track of the properties of a spline signal and overload common operators.

The representation is dimensionality-independent, and much of the code is vectorized.

Units tests are included, these require the MATLAB xunit toolbox.

[1] M. Unser, A. Aldroubi, M. Eden, "B-Spline Signal Processing: Part I-Theory", IEEE Transactions on Signal Processing, vol. 41, no. 2, pp. 821-833, February 1993

[2] M. Unser, A. Aldroubi, M. Eden, "B-Spline Signal Processing: Part II-Efficient Design and Applications", IEEE Transactions on Signal Processing, vol. 41, no. 2, pp. 834-848, February 1993

[3] M.Unser, "Splines: A Perfect Fit for Signal and Image Processing", IEEE Signal Processing Magazine, vol. 16, no. 6, pp. 22-38, 1999

## Installation

To install the toolbox Unzip the archive to a location on your harddrive. Add the root directory of the installation to your matlab path. Optionally run the unit tests to check the installation (see next section.) You can also try to run this example cell file to regenerate the output.

## Running the unit tests

To run the unit tests you need the MATLAB xunit toolbox by Steve Eddings which can downloaded from http://www.mathworks.com/matlabcentral/fileexchange/22846 Simply enter the test directory and run xunit on the MATLAB command line by typing runtest. For details, see the xunit documentation.

## Introductory example

We can create an instance of the Bspline class to approximate a signal as a sum of B-spline functions. The resulting Bspline object keeps track a few properties of the approximated signal and overloads many common operators in addition to giving the opportunity to evaluate the spline on the subscript space of the original signal.

The signal we want to approximate, defined on a uniform integer-valued grid

```
x = 1:20;
s = atan(x-10);
plot(x,s);
```

Create a third order B-spline object:

```
s_spl = Bspline(s,3);
```

We can get back a close approximation of the original signal vector by evaluating the B-spline on integer points using familiar subscripting syntax:

```
s_rec = s_spl(1:20);
plot(1:20, s_rec);
```

But it is also possible to evaluate the B-spline on a finer grid using the same syntax, effectively interpolating between the integer knots.

```
x_fine = 1:0.2:20;
s_fine_rec = s_spl(x_fine);
plot(x_fine, s_fine_rec);
```

## Approximaton accuracy

How well does this approximate the original arctangent function?

```
s_fine = atan(x_fine-10);
plot(x_fine, s_fine-s_fine_rec);
grid on; grid minor;
```

This is an exactly interpolating spline, so the error is zero at the integer knots, but higher between.

Maybe a higher spline order can improve the approximation? Or in this case maybe we are better of by using denser sampling. Let us try a fifth order spline.

```
s_spl5 = Bspline(s, 5);
s_fine_rec = s_spl5(x_fine);
plot(x_fine, s_fine-s_fine_rec);
grid on; grid minor;
```

That did not help much, actually it looks a little worse. Let us instead try to increase the sample density by a factor of 10.

```
x10 = 1:200;
x10_fine = 1:0.2:200;

s10 = atan((x10/10)-10);
s10_fine = atan((x10_fine/10)-10);

s10_spl = Bspline(s10, 3);
s10_fine_rec = s10_spl(x10_fine);

plot(x10_fine, s10_fine-s10_fine_rec);
```

The approximation is better, but still there is some approximation error at the edges of the signal. one likely reason for this; the mirrored signal extension implicit in the direct transform estimation gives a discontinuity in higher order derivatives (unless the signal under approximation has moments which die off at the edge). Since high order B-splines model continous higher order derivatives and the signal extension in this case gives discontinous higher order derivatives on the edge, there is not such a good fit.

## Coefficient characteristics

Let us look at the B-spline coefficients themselves:

```
stem(s_spl.c); title('samplerate 1, 3rd order')
```

```
stem(s10_spl.c); title('samplerate 10, 3rd order')
```

```
stem(s_spl5.c); title('samplerate 1, 5th order')
```

We can see that the coefficients are in same range as the original vector regardless of order and sampling density in this example.

## Derivatives and integration

Once the approximation has been done during construction, the B-spline can be exactly derived or integrated. For derivatives use the overloaded diff function:

```
s10_spl_diff = diff(s10_spl);
s10_diff_rec = s10_spl_diff(x10_fine);
plot(x10_fine, s10_diff_rec);
```

The corresponding integral function:

```
s10_spl_int = integral(s10_spl);
s10_int_rec = s10_spl_int(x10_fine);
plot(x10_fine, s10_int_rec);
```

How well is the approximation for the differentiation here? The arctanget has an analytic differential which we can compare against.

```
s10_diff = (1/10)./(1+(x10_fine/10-10).^2);
plot(x10_fine, s10_diff_rec-s10_diff);
```

The edge errors from the first approximation is propagated, otherwise it looks quite good. Compare this with simply using the difference operator on the input vector:

```
s10_diff2 = diff(s10);
s10_diff = s10_spl_diff(x10);
plot(x10(1:end-1),s10_diff2-s10_diff(1:end-1));
```

## 2D splines

The toolbox also lets you work on multi-dimensional splines. We can create a Bspline object for a 2d array:

```
fun2d = peaks(32);
sfun2d = Bspline(fun2d,3);

surf(double(sfun2d));
```

For such splines we can also calculate the gradient:

```
grad_fun2d = gradient(sfun2d);
quiver(grad_fun2d{2},grad_fun2d{1});
```

Published with MATLAB® 7.11
