# Supplementary material for: Joint Registration and Conformal Prediction for Partially Observed Functional Data
Source: J Comput Graph Stat. Author manuscript; Available in PMC 2026 May 12. (PMC13160368; doi:10.1080/10618600.2026.2634823)
Supplement: Supp 1 [file NIHMS2168257-supplement-Supp_1.zip › JointRegistrationConformalPrediction_MATLAB2022b.zip!/JointRegistrationConformalPrediction_MATLAB2022b/jdtuck-fdasrvf_MATLAB-3.6.3.0/armadillo_cpp/armadillo/mex_interface/armaMex_documentation.pdf]

armaMex connector documentation. Version 1.0.0

Variables and constants:

Type: type is the C++ data type (int, float, double…)

mxArray: mxArray is the pointer to the input matrices from Matlab – prhs[k] or the output matrices to Matlab – plhs[k].

mxComplexity: Matlab complexity flag. Used when creating Matlab matrices. Real matrices use mxREAL and complex matrices use mxCOMPLEX flag.

mxClassID: Matlab class type. Used when creating Matlab matrices.

mxClassID Value	MATLAB Type	MEX Type	C Primitive Type
**mxINT8_CLASS**	int8	int8_T	char, byte
**mxUINT8_CLASS**	uint8	uint8_T	unsigned char, byte
**mxINT16_CLASS**	int16	int16_T	short
**mxUINT16_CLASS**	uint16	uint16_T	unsigned short
**mxINT32_CLASS**	int32	int32_T	int
**mxUINT32_CLASS**	uint32	uint32_T	unsigned int
**mxINT64_CLASS**	int64	int64_T	long long
**mxUINT64_CLASS**	uint64	uint64_T	unsigned long long
**mxSINGLE_CLASS**	single	float	float
**mxDOUBLE_CLASS**	double	double	double

Functions:

Type armaGetScalar(const mxArray *matlabScalar)

Get the real valued scalar from Matlab. If the input is a matrix or a cube the function will return the real valued first entry of the matrix M(1,1) or the cube C(1,1,1).

```
Example:
int value = armaGetScalar(prhs[0]);
```

double armaGetDouble(const mxArray *matlabScalar)

Get the real double valued scalar from Matlab. If the input is a matrix or a cube the function will return the real valued first entry of the matrix M(1,1) or the cube C(1,1,1).

```
Example:

double value = armaGetScalar(prhs[0]);
```

Mat<Type> armaGetData(const mxArray *matlabMatrix,bool copy_aux_mem = false, bool strict = true)

Get the real part of a Matlab matrix. By default the allocated memory in Matlab is used for faster access. If the matrix size or shape will be changed copy_aux_mem should be set to true.

```
Example:

Mat<int> M = armaGetData<int>(prhs[0]);

Mat<int> M_Copy = armaGetData<int>(prhs[0], true);
```

Mat<double> armaGetPr(const mxArray *matlabMatrix,bool copy_aux_mem = false, bool strict = true)

Get the real double part of a Matlab matrix. This definition is used in conjunction with the Matlab mex definition of mxGetPr.

```
Example:

mat M = armaGetPr(prhs[0]);

mat M_Copy = armaGetPr(prhs[0], true);
```

Mat<Type> armaGetImagData(const mxArray *matlabMatrix,bool copy_aux_mem = false, bool strict = true)

Get the imaginary part of a Matlab matrix. By default the allocated memory in Matlab is used for faster access. If the matrix size or shape will be changed copy_aux_mem should be set to true.

```
Example:

Mat<float> M = armaGetImagData<float>(prhs[0]);

Mat<float> M_Copy = armaGetImagData<float>(prhs[0], true);
```

Mat<double> armaGetPi(const mxArray *matlabMatrix,bool copy_aux_mem = false, bool strict = true)

Get real double part of a Matlab matrix. This definition is used in conjunction with the Matlab mex definition of mxGetPi.

```
Example:
mat M = armaGetPi(prhs[0]);
mat M_Copy = armaGetPi(prhs[0], true);
```

cx_mat armaGetCx(const mxArray *matlabMatrix,bool copy_aux_mem = false, bool strict = true)

Get the complex matrix from Matlab. . By default the allocated memory in Matlab is used for faster access. If the matrix size or shape will be changed copy_aux_mem should be set to true.

```
Example:
cx_mat M = armaGetCx(prhs[0]);
cx_mat M_Copy = armaGetCx(prhs[0], true);
```

void armaSetData(mxArray *matlabMatrix, const Mat<Type>& armaMatrix)

Return the Armadillo matrix armaMatrix to Matlab as the real part of the matrix. The Matlab matrix must first be created using armaCreateMxMatrix.

```
Example:
mat A = randu<mat>(5,6);
plhs[0] = armaCreateMxMatrix(A.n_rows,A.n_cols);
armaSetData(plhs[0],A);
```

void armaSetPr(mxArray *matlabMatrix, const Mat<double>& armaMatrix)

Return the double valued Armadillo matrix armaMatrix to Matlab as the real part of the matrix. The Matlab matrix must first be created using armaCreateMxMatrix.

```
Example:
mat A = randu<mat>(5,6);
plhs[0] = armaCreateMxMatrix(A.n_rows,A.n_cols);
armaSetPr(plhs[0],A);
```

void armaSetImagData(mxArray *matlabMatrix, const Mat<Type>& armaMatrix)

Return the Armadillo matrix armaMatrix to Matlab as the imaginary part of the matrix. The Matlab matrix must first be created using armaCreateMxMatrix.

```
Example:
mat A = randu<mat>(5,6);
plhs[0] = armaCreateMxMatrix(A.n_rows,A.n_cols);
armaSetImagData(plhs[0],A);
```

void armaSetPi(mxArray *matlabMatrix, const Mat<double>& armaMatrix)

Return the double valued Armadillo matrix armaMatrix to Matlab as the imaginary part of the matrix. The Matlab matrix must first be created using armaCreateMxMatrix.

```
Example:
mat A = randu<mat>(5,6);
plhs[0] = armaCreateMxMatrix(A.n_rows,A.n_cols);
armaSetPi(plhs[0],A);
```

void armaSetCx(mxArray *matlabMatrix, const cx_mat& armaMatrix)

Return the complex Armadillo matrix armaMatrix to Matlab as a complex matrix. The Matlab matrix must first be created using armaCreateMxMatrix as a complex matrix with mxComplexity flag set to mxCOMPLEX.

```
Example:
mat A = randu<mat>(5,6);
mat B = randn<mat>(5,6);
cx_mat C(A,B);
plhs[0] = armaCreateMxMatrix(A.n_rows,A.n_cols,mxDOUBLE_CLASS,mxCOMPLEX);
armaSetPi(plhs[0],C);
```

Cube<Type> armaGetCubeData(const mxArray *matlabMatrix,bool copy_aux_mem = false, bool strict = true)

Get the real part of the 3-dimensional Matlab matrix. By default the allocated memory in Matlab is used for faster access. If the cube size or shape will be changed copy_aux_mem should be set to true.

```
Example:
Cube<int> C = armaGetCubeData<int>(prhs[0]);
```

Cube<double> armaGetCubePr(const mxArray *matlabMatrix,bool copy_aux_mem = false, bool strict = true)

Get the real double part of the 3-dimensional Matlab matrix. By default the allocated memory in Matlab is used for faster access. If the cube size or shape will be changed copy_aux_mem should be set to true.

```
Example:
cube C = armaGetCubePr(prhs[0]);
```

Cube<Type> armaGetCubeImagData(const mxArray *matlabMatrix,bool copy_aux_mem = false, bool strict = true)

Get the imaginary part of the 3-dimensional Matlab matrix. By default the allocated memory in Matlab is used for faster access. If the cube size or shape will be changed copy_aux_mem should be set to true.

```
Example:
Cube<float> C = armaGetCubeImagData<float>(prhs[0]);
```

Cube<double> armaGetCubePi(const mxArray *matlabMatrix,bool copy_aux_mem = false, bool strict = true)

Get the imaginary double part of the 3-dimensional Matlab matrix. By default the allocated memory in Matlab is used for faster access. If the cube size or shape will be changed copy_aux_mem should be set to true.

```
Example:
Cube C = armaGetCubePi(prhs[0]);
```

void armaSetCubeData(mxArray *matlabMatrix, const Cube<Type>& armaCube)

Return the Armadillo cube armaCube to Matlab as the real part of the 3-dimensional matrix. The Matlab matrix must first be created using armaCreateMxMatrix.

```
Example:
cube C = randu<mat>(5,6,7);
plhs[0] = armaCreateMxMatrix(A.n_rows,A.n_cols,A.n_slices);
armaSetCubeData(plhs[0],A);
```

void armaSetCubePr(mxArray *matlabMatrix, const Cube<double>& armaCube)

Return the double valued Armadillo matrix armaCube to Matlab as the real part of the 3-dimensional matrix. The Matlab matrix must first be created using armaCreateMxMatrix.

```
Example:
cube C = randu<mat>(5,6,7);
plhs[0] = armaCreateMxMatrix(A.n_rows,A.n_cols,A.n_slices);
armaSetCubePr(plhs[0],A);
```

void armaSetImagCubeData(mxArray *matlabMatrix, const Cube<Type>& armaCube)

Return the Armadillo cube armaCube to Matlab as the imaginary part of the 3-dimensional matrix. The Matlab matrix must first be created using armaCreateMxMatrix.

```
Example:
cube C = randu<mat>(5,6,7);
plhs[0] = armaCreateMxMatrix(A.n_rows,A.n_cols,A.n_slices);
armaSetImagCubeData(plhs[0],A);
```

void armaSetCubePi(mxArray *matlabMatrix, const Cube<double>& armaCube)

Return the double valued Armadillo matrix armaCube to Matlab as the imaginary part of the 3-dimensional matrix. The Matlab matrix must first be created using armaCreateMxMatrix.

```
Example:
cube C = randu<mat>(5,6,7);
plhs[0] = armaCreateMxMatrix(A.n_rows,A.n_cols,A.n_slices);
armaSetCubePi(plhs[0],A);
```

void armaSetCubeCx(mxArray *matlabMatrix, const cx_cube& armaCube)

Return the complex double valued Armadillo armaCube to Matlab as a complex 3-dimensional matrix. The Matlab matrix must first be created using armaCreateMxMatrix as a complex matrix with mxComplexity flag set to mxCOMPLEX.

```
Example:
cube A = randu<mat>(5,6,7);
cube B = randn<mat>(5,6,7);
cx_cube C(A,B);
plhs[0] = armaCreateMxMatrix(A.n_rows,A.n_cols,A.n_slices,mxDOUBLE_CLASS,mxCOMPLEX);
armaSetCubePi(plhs[0],C);
```

mxArray* armaCreateMxMatrix(const mwSize n_rows,const mwSize n_cols,const mxClassID mx_type = mxDOUBLE_CLASS,const mxComplexity mx_complexity = mxREAL)

Creates the 2-Dimensional Matlab matrix to be used as output.

```
Example:
plhs[0] = armaCreateMxMatrix(A.n_rows,A.n_cols);
plhs[1] = armaCreateMxMatrix(A.n_rows,A.n_cols,mxSINGLE_CLASS,mxREAL);
plhs[2] = armaCreateMxMatrix(A.n_rows,A.n_cols,mxDOUBLE_CLASS,mxCOMPLEX);
```

mxArray* armaCreateMxMatrix(const mwSize n_rows, const mwSize n_cols, const mwSize n_slices, const mxClassID mx_type = mxDOUBLE_CLASS, const mxComplexity mx_complexity = mxREAL)

```
Example:
plhs[0] = armaCreateMxMatrix(A.n_rows,A.n_cols,A.n_slices);
plhs[1] = armaCreateMxMatrix(A.n_rows,A.n_cols,A.n_slices,mxSINGLE_CLASS,mxREAL);
plhs[2] = armaCreateMxMatrix(A.n_rows,A.n_cols,A.n_slices,mxDOUBLE_CLASS,mxCOMPLEX);
```

SpMat<Type> armaGetSparseData(const mxArray *matlabMatrix,bool sort_locations = false)

Get the real part of a sparse matrix from Matlab. Note that the matrix must be converted to sparse in Matlab (for example using sparse(matrix) command). Currently Matlab only supports sparse matrices of type double and logical (Boolean).

```
Example:
SpMat<double> Sparse = armaGetSparseData<double>(prhs[0]);
SpMat<double> Sparse = armaGetSparseData<double>(prhs[0], true);
```

SpMat<double> armaGetSparseMatrix(const mxArray *matlabMatrix,bool sort_locations = false)

Get the double valued real part of a sparse matrix from Matlab. Note that the matrix must be converted to sparse in Matlab.

```
Example:
sp_mat Sparse = armaGetSparseMatrix(prhs[0]);
sp_mat Sparse = armaGetSparseMatrix(prhs[0], true);
```

SpMat<Type> armaGetSparseImagData(const mxArray *matlabMatrix,bool sort_locations = false)

Get the imaginary part of a sparse matrix from Matlab. Note that the matrix must be converted to sparse in. Currently Matlab only supports sparse matrices of type double and logical (Boolean).

```
Example:
SpMat<double> Sparse_Imag = armaGetSparseImagData<double>(prhs[0]);
SpMat<double> Sparse_Imag = armaGetSparseImagData<double>(prhs[0], true);
```

SpMat<double> armaGetSparseImagMatrix(const mxArray *matlabMatrix,bool sort_locations = false)

Get the double valued imaginary part of a sparse matrix from Matlab. Note that the matrix must be converted to sparse in Matlab.

```
Example:
sp_mat Sparse = armaGetSparseImagMatrix(prhs[0]);
sp_mat Sparse = armaGetSparseImagMatrix(prhs[0], true);
```

void armaSetSparsePr(mxArray *matlabMatrix, const SpMat<double>& armaMatrix)

Return the sparse matrix armaMatrix as the real part of the sparse Matlab matrix. The sparse Matlab matrix must be created using armaCreateMxSparseMatrix.

```
Example:
plhs[0] = armaCreateSparseMatrix(Sparse.n_rows,Sparse.n_cols,Sparse.n_nonzero);
armaSetSparsePr(plhs[0],Sparse);
```

void armaSetSparsePi(mxArray *matlabMatrix, const SpMat<double>& armaMatrix)

Return the sparse matrix armaMatrix as the imaginary part of the sparse Matlab matrix. The sparse Matlab matrix must be created using armaCreateMxSparseMatrix.

```
Example:
plhs[0] = armaCreateSparseMatrix(Sparse.n_rows,Sparse.n_cols,Sparse.n_nonzero,mxCOMPLEX);
armaSetSparsePr(plhs[0],Sparse);
armaSetSparsePi(plhs[0],Sparse_Imag);
```

mxArray* armaCreateMxSparseMatrix(const mwSize n_rows,const mwSize n_cols,const mwSize n_nonzero,const mxComplexity mx_complexity = mxREAL)

Create a sparse matrix in matlab.

```
Example:
```

```
plhs[0] = armaCreateSparseMatrix(Sparse.n_rows,Sparse.n_cols,Sparse.n_nonzero);
```
